# Essay on the Functions of the Nervous System

**Published:** 1815-04

**Authors:** C. W. Smerdon

**Affiliations:** Clifton, Bristol


### *For the Medical and Physical Journal.*

### *Essay on the Functions of the Nervous System;* by Mr. C. W. Smerdon.

*(Continued from p. 88.)*

TO the eye of an uninformed observer, the blood, as it circulates in the vessels, would appear perfectly homogeneous; nor would any argument convince him, that so many different compound fluids as we find in the animal structure, are formed out of this one and apparently simple substance.

Out of the body, the blood is seen in three different states: fluid, as it flows from the vessels; solid, after standing a few minutes; and lastly, fluid and solid, when the crassamentum is separated from the serum. These processes, which art has not hitherto been able to prevent, are not less remarkable because they are common. The opinion of the celebrated Hunter on the vitality of the blood, explains very satisfactorily the cause of its coagulation; for blood, when out of the vessels, is placed in an unnatural situation, and, as it can be no longer subservient to the purposes for which it was originally intended, it consequently must lose the principle of life which blood possesses in common with every other part of an organized body; and, if (as Mr. Hunter has clearly proved) the fluidity of the blood be dependant on its containing that principle, then, it is clear, that the loss of one must be followed by a loss of the other, and that death and coagulation are connected together in the relation of cause and effect.

Mr. Hunter, however, was not content with satisfactorily clearing up this curious physiological enigma, but he went on to the establishment of an hypothesis, which he has founded on principles that are, in my opinion, erroneous. He considered the blood as being the grand seat or centre of life, from whence every other part of the body derived its vitality; and, in support of the opinion, he has adduced the well-known fact, that mortification always takes place in a limb, when, from some cause or other, its supply of blood has been completely cut off. This argument is specious, but it is not conclusive; for it is proved, by the contractions which the muscles of an apparently dead animal may be thrown into by galvanism, that the muscular fibres at least retain their vitality for some time after the circulation of the blood and the motion of the heart have ceased for ever; indeed, it is the first proof which we have of the absolute death of an animal, when he is no longer sensible to the impression of that powerful stimulus. The cessation of the living living actions of a part, which are carried on by the two great systems of the body, is synchronous with the apparent cessation of life; for, although the living principle be not primarily dependant on actions, it soon ceases to be inherent in a disorganized part, where actions have consequently ceased; and it is evident, that a limb, in which the circulation of the blood has ceased, is exactly placed in such a situation.

But it is assumed as a fact, by some who strictly adhere to this hypothesis of Mr. Hunter, that the functions of the sanguiferous system are carried on independent of the nervous; and, indeed, it seems necessary for them to adopt this opinion, for on no other ground could they assert that the seat of life is not in the brain as well as in the blood. It may be said, that a paralytic limb not only preserves its vitality, but that increased action may be excited in it; this, however, is a very weak argument, unless it could be proved that under such circumstances the supply of nervous energy is entirely cut off. It is, indeed, evident, that Nature never formed any thing in vain; why then, I will ask, are the secretory organs endowed with nerves? not, certainly, with the view of communicating motion to them, for, with respect to this, they are all passive; nor with the intention of exciting the calorific functions, nor of assisting in the glandular secretions, if it be assumed, as a fact, on the one hand, that the Priestleian doctrine of animal heat is correct; and, on the other, that the functions of the arterial system are carried on independent of the nervous. It is improbable also, that the sole intention of nerves, which are sent to the internal structure of glands, is to furnish them with sensation, because they are wholly independent of the will, and surely feeling is a very secondary, if at all a necessary, object to parts which are protected by considerable coverings from external violence. Hence, then, we have no other means of satisfactorily answering that question, than by admitting, that the functions of the sanguiferous system are not carried on independent of the brain; and, therefore, it must follow, as a necessary consequence, that a living solid cannot be produced from the blood, without an equal participation of the nervous energy. To illustrate this subject, let us take as an example a portion of coagulable lymph at the instant that it is secreted from the vessels, and which I will presently still further attempt to demonstrate, is the product of a reciprocal action between the nervous and arterial extremities. Now, are we to suppose, that this matter is not, at the precise period which I have mentioned, endowed with vitality?

lity? Is it not, in fact, a living solid, being the product of a living action? the answer must be in the affirmative, and still it is clear, that the endowment of this new part with arteries and nerves, or, in other words, its becoming organized, is a subsequent process; but it will be here said, that this lymph is effused or secreted from the blood, which, being the seat of life, gives vitality to whatsoever is formed from it. To this, I will answer, that as it cannot be formed without the presence and co-operation of nerves, so it is as fair to infer, that life proceeds from the brain, through the medium of the nerves, as from the blood through the medium of the vessels; for, when blood is extravasated, it immediately loses its vitality and becomes solid, and it must be by a process, entirely out of the common course of nature, to infuse life into it again, so as to become a proper receptacle or bed for vessels, nerves, and absorbents, to shoot into, and thus be transformed from a dead to a living organized solid, and be enabled to live by its own inherent powers. If, however, the vitality of all parts of the body be supported by the blood alone, from whence does this fluid in its turn receive life? for, since the body is constantly going to decay, and as constantly renewing, it is evident that the blood would soon be exhausted of its living powers, if there were not a source from whence even this may become replenished. Do the absorbent vessels carry back into the circulating system the living principle which has been separated from the worn-out parts of the body? this opinion, if adopted, would be highly ridiculous, because life is immaterial and superadded to structure. Is the blood replenished with life, as well as with its own tangible matter, from the chyle? this also is untenable, because chyle is formed out of dead matter, and owes its vitality to the change which the food undergoes by the living actions of the stomach and duodenum. Upon the whole, then, it is highly probable, that, as life is superadded to structure, so it is not particularly contained in this or that portion of an organized body, but is diffused throughout the whole; and, as blood when thrown out of the vessels, coagulates and dies, while the matter which forms the solids of the body also coagulates but lives, so I will conclude that this vital coagulated matter, which differs so essentially from coagulated blood, is the product of a reciprocal action between the arterial and nervous systems, in conjunction with the living principle; and from this action alone it receives its vitality.

Life then may be defined to be an unknown principle which pervades all matter that is susceptible of increase by

 growth growth. It is this principle which first developes the actions of the seeds of plants, and directs the radicle to shoot downward into the earth, and the plumula to take a contrary course; and it can be no other than this living intelligence which directs the slender twigs of the bean-tree to entwine for support around the nearest prop. The actions connected with animal or vegetable existence are derived from the mechanism of structure, and are more or less numerous, simple, or intricate, in proportion as the structure is more or less complicated. The living principle is immaterial and simple, and precisely the same in vegetables and in animals. It is not the sole cause, and certainly not the effect of actions; but it may be said to direct them, in the same manner as an artisan directs the operations of a piece of machinery.

The blood, then, possesses vitality only so far as it forms a part of a living whole. When it is extravasated or thrown out of the vessels, being disjoined from that whole for ever, it consequently dies, and is mingled with the common matter of the universe. In like manner, when the actions of a part cease, in consequence of the want of blood or nervous energy, the harmony between the part and the whole being by this means destroyed, it loses its vitality, because life can no longer co-operate towards the production of actions which seem to be absolutely necessary for the union of an organized body. The living powers of the whole throws off this dead part, and it becomes common matter.

Thus have I presumed to dissent from the opinion of a man whose name will always be considered as the brightest ornament of his profession; opinions, too, which have long been held sacred because they were his. But it is necessary that I should do so, in order to give that degree of importance to the nervous system, which I am convinced it is entitled to: nor is it possible to form a rational theory on the process of secretion, if at the same time it be considered a fact, that the blood and the nerves do not act conjointly upon each other.

Every secreting gland, we know, is furnished with similar materials, namely, an artery, a nerve, and an excretory duct, and also with the same matter to work upon; and still every class of these not only produce a compound fluid peculiar to themselves, but chemistry has ascertained, that one of the constituent parts of some of these fluids is not to be found in the minutest analysis of the blood. Thus, in the bile we have the biliary matter of Berzelius, in the urine we have the urea; and Dr. Bostock informs us, that the mucous fluid differs from the albuminous in being principally composed of a suba substance which is not exactly similar to any thing in the blood.

Secretion has always been considered as a chemical process, and consequently authors, who have written on this subject, have attempted to explain it in conformity with the then-existing state of chemical knowledge. The celebrated Baron Haller, in his no-less celebrated book on Physiology, has endeavoured to explain this secret work of nature conformably to rules which his predecessors had followed. In his time (when compared with the present) the modes of analysing bodies were very limited and fallacious, being chiefly confined to distillation, evaporation, roasting, &c. By the first of these processes the blood was considered to be divided into its elements, namely water, salt, earth, and a highly empereumatic oil; which last (probably the mere creature of the fire) was the residuary matter after the two first were driven off. Assuming as a fact that the blood became thus decomposed in its passage through or towards the secreting organs, he thought that some peculiarity in the form, diameter, or length, of the excretory ducts, as also a difference in the tenacity and specific gravity of the elementary parts of the blood, were sufficient to explain the phenomenon of secretion, by allowing only particular elements to pass through a particular duct or set of ducts. The adoption of reagents for the analysing of compound bodies ranks among the most important discoveries of modern chemistry. By the agency of these, the component parts of bodies have been accurately defined, and the list of simple substances considerably lessened. Notwithstanding, it is a self-evident principle, that a fluid, when changed into another, must first become decomposed; and, in proportion as this decomposition is partial or complete, must the new fluid lose the characteristic properties of its parent. The relation between the artery, vein, and excretory duct, of a secreting organ will not admit of a belief, that blood, previous to its becoming transformed into another fluid, is decomposed through the medium of a reagent, for an artery, on entering a gland, divides and subdivides into the minutest ramifications; these, at a certain part, after giving off another set of vessels (the excretory ducts), pass onward without any other distinguishing mark as to where arteries end and veins begin.

*Clifton, Bristol;* C. W. SMERDON.

*Feb.* 16, 1815.

*(To be continued.)*

